# Editorial: Gene regulation in lymphocyte development and response

**DOI:** 10.3389/fimmu.2026.1885781

**Published:** 2026-06-11

**Authors:** Ananda L. Roy, Ranjan Sen, Dinah S. Singer

**Affiliations:** 1 National Institute on Aging, Laboratory of Molecular Biology and Immunology, National Institutes of Health, Bethesda, MD, United States; 2 Experimental Immunology Branch, Center for Cancer Research, NCI, National Institutes of Health, Bethesda, MD, United States

**Keywords:** B cells, gene regulation, immune system, T cells, transcription factors

## Introduction

Before the advent of modern life saving medicines, the immune system protected human beings from deadly pathogens and foreign objects for thousands of years. Given that the diversity of pathogens and microbes far outnumber human beings, it is no wonder that the immune system had to evolve extremely intricate and elaborate ways to fight these infectious agents and keep our species alive (1). Although we have come a long way to begin to understand the complexities of our immune system and its regulation in health and disease, it is fair to state that our understanding is far from complete (2–4). We now know that the first level of immune protection is provided by the innate immune system, that it is a rapid response and conserved from lower to higher eukaryotes. Adaptive immunity, by contrast, is restricted to vertebrates, providing a focused, more specific, although slower, response to clear the infectious agents. Even though these pathways of immune responses are distinct, there are cells (e.g., marginal zone B cells and NKT cells) and molecules (e.g., NFκB) that play a sentinel role between these two systems (2–4). Addressing both arms of the immune system at once is daunting and beyond the scope of this Research Topic. Hence, our special topic was designed primarily to address the molecular regulation and pathways of the adaptive immune system. Even more specifically, we focused on gene regulatory pathways in B and T lymphocytes.

Transcriptional regulation of gene expression is a complex and coordinated process, requiring hundreds of factors to converge on a target gene in a cell type and time-dependent manner in response to a specific physiological cue (5). When transitioning from a dormant to an active state, specific biological cues (such as encounter with pathogens) are transduced within the cell through cell surface receptors and are read by the cellular machinery to direct them to target genes (5, 6). In addition to direct involvement of transcription factors in signaling process (like NFκB and STAT proteins), indirect regulation of genes involved in signal transduction (e.g., by MIZ-1) is also observed. It needs to be appreciated that the 2M long chromatin is highly compacted in time and space to fit into the 10μM nuclear space with topologically separated domains that are associated with heterochromatin (or B domain, where silenced genes often reside) and euchromatin (or A domain, containing active genes) (7). To activate specific genes, these domains/regions often need to be topologically altered (e.g., through loop extrusion by anchoring protein like CTCF or even by non-coding RNAs) depending on the physiologic signal allowing gene transcription to ensue (7). An added complexity in antigen receptor loci is that they need to be rearranged (as during VDJ recombination) prior to expression (8). As we know, native chromatin is decorated with post-translationally modified histone proteins and DNA modifications that together define cellular epigenetic states (9, 10). Specialized “reader” and “writer” proteins can rewrite/reorganize these epigenetic marks (e.g., methylate or demethylate DNA by TET enzymes) as and when needed to activate transcription. Once the stage is set for transcription to occur, a multitude of transcription factors and co-factors (like OCA-B and TOX) are recruited to the target gene to promoter proximal or distal (enhancer) regions to ensure both fidelity and accuracy of transcription are achieved in time and space. During this process, not only transcriptional activators but repressors often play crucial roles (e,g., Bach2). It is thus evident from this multi-step, highly coordinated process that any miscommunication or even a slight misalignment can have profound and devastating effects both at cellular and at organismal levels leading to disease. The articles in our Research Topic illustrate many of these molecular steps and exemplify genetic experiments to validate them.

Our intent was to keep this topic broad enough to invite, reviews, opinion pieces and original research work. Accordingly, we are pleased to note that seven articles report primary research results and 3 are reviews and opinion pieces. Out of the ten articles in this section, four are focused on regulation in B lymphocytes and six (one on iNKT) are on T lymphoid cell types. Together these articles illustrate the incredible complexities of the immune system and the varied approaches and toolbox at our disposal to address these complexities. Below we outline highlights from each of these articles.

## Epigenetic regulation of gene expression

Changes in gene expression in response to immune modulation often begin epigenetically. Rao and colleagues examine how transcription factor FOXP3, found in T regulatory cells, regulates immune balance and effector function. More specifically, they test mechanistically how the expression of *Foxp3* gene is often lost when Tregs switch to “ex-Treg” cells that are associated with pathology under strong or chronic inflammatory conditions. One way this could be achieved is via enhanced methylation of CNS1 and CNS2 enhancers in the Foxp3 locus. Normally these enhancers remain in an open/demethylated state mediated by TET enzymes. Genetic ablation of *Tet2/3* in mice results in increased methylation of the enhancers and consequently, increased conversion of Treg cells to their pathological version. Interestingly, base-resolution sequencing showed that in addition to direct increase in methylation/silencing of *Foxp3* enhancers due to TET deficiency, the observed gene expression patterns might also result from interference with binding of methylation-sensitive transcriptional repressors including CTCF.

TET2/3 enzymes also play an important epigenetic regulatory function in cells other than Tregs. Tsagaratou and colleagues show they are important for thymic invariant natural killer T (iNKT) cells. Loss of Tet2/3 results in upregulation of several genes in iNKT cells due to the loss of repressor proteins that keep gene expression in check. DROSHA, that is essential for microRNA biogenesis, is among these repressors. Absence of TET2/3 enzymes suppress expression of *Drosha* by enhanced methylation across the gene body and thereby limiting chromatin accessibility. As a result, expression of the key microRNA family, let7, is diminished and expression of its target iNKT cell specific transcription factor PLZF is also reduced.

## Locus accessibility

The immunoglobulin receptor loci in B cells undergo rearrangement before they are transcribed, a step that involves differential use of variable region segments (V_H_). This process is still incompletely understood as some distal V segments are used in preference to proximal V segments. The Opinion/Hypothesis piece by Kenter and colleagues proposes an interesting and integrated model of how long non-coding RNAs (lncRNAs) (11), which regulate chromatin architecture, might be involved in determining why some distal segments join in preference over proximal segments to generate a diverse IgH repertoire during successful V(D)J recombination of the B cell receptor. Their model is supported in part by the fact that the expression of lncRNAs is highly elevated at the IgH and other receptor loci involved in V(D)J recombination. Thus, in addition to loop-extrusion, lncRNAs might play a critical role in B cell receptor recombination events.

## Transcription factor affecting signaling

One of the primary research papers on B lymphocytes comes from Moroy and colleagues. MIZ-1 is a well-established interacting partner of the cellular oncogenic product, MYC. By analyzing mice expressing a POZ domain deficient MIZ-1, they show that MIZ-1 transcription factor in addition to regulating autophagy-related genes, also regulates genes involved in B cell receptor signaling and cytoskeletal dynamics. Thus, defective MIZ-1 results in decreased survival of follicular (but not marginal zone) B cells. Given that MIZ-1 interacts with oncogenic factor MYC, the expansion of MYC-dependent B cell lymphomas are impaired in mice expressing defective MIZ-1.

## Immune regulation via enhancer actions

Development of T cells in the thymus proceed through multiple stages. Thymocytes fall into three main classes: double negative (DN, CD4^-^CD8^-^), double positive (DP, CD4^+^CD8^+^) or single positive (SP, either CD4^+^CD8^-^ or CD4^-^CD8^+^). The SP stage can further be subdivided as intermediate (INT, CD4^+^CD8^lo^) or immature single positive (ISP, CD8+CD4-). Sarafova and colleagues address the regulation of *Cd4* gene during thymic development, specifically focusing on a conserved region known as NCE/E4m that is proximal to the Cd4 silencer but contains essential developmental stage specific enhancer properties. Through both *in vitro* and *in vivo* (transgenic mice) experiments they demonstrate that NCE/E4m is critical for enhanced CD4 expression following positive selection in a stage specific fashion: it is required at the INT stage but not at the DP stage.

Although the T cell receptor gene expression is crucial for thymic identity and mounting an effective immune response, the precise regulation of TCR genes is still under investigation. Buchholtz and colleagues address regulation of the TCRα chain gene in mature peripheral T cells. They show via a pooled CRISPR screen methodology that in contrast to the 5’J region, the Tα2 region is the primary driver of TCRα chain expression in a subset of mature peripheral T cells. This could have ramifications for future CAR T cell therapies.

## Transcription factors and co-factors directly involved in immune gene regulation

The review by Roeder and Lu focuses on the first identified cell type specific coactivator, OCA-B (OCT Coactivator from B cells). Although originally characterized as a biochemical entity, elaborate genetic studies mostly in murine systems, firmly delineates its essential role in germinal center (GC) formation as well as in regulation of bone marrow and peripheral B cell development. OCA-B transcriptionally regulates IgH, Bcl6, Mef2b and Irf4 genes. Importantly, this review also describes OCA-B’s role in regulating gene expression in follicular T helper cells (which is further addressed by Tantin and colleagues), thus making it a comprehensive and timely review for the field.

Although as stated above, the transcriptional coactivator OCA-B (encoded by *Pou2af1*) was first identified in B cells, it is also expressed in T cells. In attempting to address OCA-B’s mechanistic role in T cells, Tantin and colleagues deployed a series of biochemical, immunological, and genomic screening experiments. They demonstrated that OCA-B directly associates with GATA3 in T cells and represses transcription of key cytokine genes, including Il4, Il5, Il13, and Irf4 thus restricting Th2 function. Using an *in vivo* murine papain allergy model they further show that OCA-B expression in T cells could limit the frequency of T cells in the lung.

B-1 cells constitute a distinct lineage found within the peritoneal cavity and thus distinguishing them from conventional B (or B-2 cells which are located within lymphoid organs such as spleen, lymph nodes and bone marrow). Functionally, B-1 cells are innate-like cells that help mount T-independent immune response, while B-2 cells mount T-dependent immune responses. The primary research paper from Youn and colleagues addresses transcriptional regulation during B-1 B cell development. Bach2 is a transcriptional repressor that is important for maintenance of B-1 cells. They show by a B-cell specific ablation of Bach 2 in mice that a self-autonomous function of Bach2 is essential for B-1 cell self-renewal in peripheral tissues but not in fetal liver or bone marrow.

Hogan and colleagues present a comprehensive review on transcription factor TOX, which has primary role in CD8+ T cell exhaustion, although it has a broader role in diverse immune cells. The authors pose the question of what might be the mechanistic basis for such diversity? Given that TOX belongs to HMG-box binding class of transcription factors, which are often called “structural” transcription factors, because they bind to and bend DNA, the authors draw interesting mechanistic parallels with more ubiquitous HMG-box binding factors and how these structural factors bind target genes and influence diverse biological functions. They finally suggest future experimental directions to mechanistically address how TOX regulates T cell exhaustion.

It is clear from this set of articles that the regulation of gene expression in immunity is an extremely complex and multistep process. Given that the adaptive immune response must be highly measured and controlled, it is expected to be so tightly regulated. However, like the adaptive immune response, the innate response is also regulated by both transcriptional and intercellular mechanisms. The diverse set of tools and technologies applied in these studies illustrate the strategies we are embarking upon to address this complex problem. The future holds much promise, and exciting avenues are bound to be further developed to continue our quest to solve the mysteries of the immune system.
